# Food Webs in Relation to Variation in the Environment and Species Assemblage: A Multivariate Approach

**DOI:** 10.1371/journal.pone.0122719

**Published:** 2015-04-16

**Authors:** Tiffany A. Schriever

**Affiliations:** Department of Ecology and Evolutionary Biology, University of Toronto, Toronto, Ontario, Canada; University of Yamanashi, JAPAN

## Abstract

The abiotic environment has strong influences on the growth, survival, behavior, and ecology of aquatic organisms. Biotic interactions and species life histories interact with abiotic factors to structure the food web. One measure of food-web structure is food-chain length. Several hypotheses predict a linear relationship between one environmental variable (e.g., disturbance or ecosystem size) and food-chain length. However, many abiotic and biotic variables interact in diverse ways to structure a community, and may affect other measures of food web structure besides food-chain length. This study took a multivariate approach to test the influence of several important environmental variables on four food-web characteristics measured in nine ponds along a hydroperiod gradient over two years. This approach allowed for testing the ecosystem size and dynamic constraints hypotheses while in context of other possibly interacting environmental variables. The relationship between amphibian and invertebrate communities and pond habitat variables was assessed to understand the underlying food-web structure. Hydroperiod and pond area had a strong influence on amphibian and invertebrate communities, trophic diversity and δ^15^N range. The range in δ^13^C values responded strongly to dissolved oxygen. Food-chain length responded to multiple environmental variables. Invertebrate and amphibian communities were structured by pond hydroperiod which in turn influenced the trophic diversity of the food web. The results of this study suggest food-chain length is influenced by environmental variation and species assemblage and that a multivariate approach may allow us to better understand the dynamics within and across aquatic food webs.

## Introduction

Temporary waters are abundant, diverse in physical, chemical, and biological characteristics, and are located throughout the world [1]. Temporary waters fall on one extreme end of the habitat duration gradient, with lentic habitats ranging from small intermittent (temporary or episodic) to large permanent habitats [2]. The hydroperiod (length of aquatic phase) is a major factor determining invertebrate and amphibian species diversity, composition, [3–5] and breeding success [3,6]. The hydroperiod gradient consists of temporary to semi-permanent ponds, to permanent ponds without fishes, to permanent ponds with fishes, and thus is characterized by variation in both abiotic (drying and associated changes in water chemistry) and biotic (increasing predation pressure) properties. The physicochemical environment of aquatic habitats is dynamic and can have severe consequences for the survival of its inhabitants. Aside from hydroperiod, water temperature is perhaps the most important abiotic factor in aquatic systems because of its effect on the growth and survival, behavior, food-web structure and ecology of aquatic organisms [7,8]. A full analysis describing the variation in aquatic communities across a hydroperiod gradient and how it pertains to food-web dynamics has not been conducted.

A current focus in food-web ecology is to understand how environmental variation influences food-web structure and function. Food webs depict consumer-resource (or predator-prey) interactions by characterizing trophic relationships among species or individuals in a particular habitat [9]. Stable isotopes are frequently used to examine the structure and function of food webs. In particular, carbon and nitrogen stable isotope ratios (δ^13^C and δ^15^N, respectively) from members of the community are plotted in bi-variate space to give an overall picture of energy flow from the base (breadth of carbon used) to the top (height of the food-web along the vertical δ^15^N axis). Typically, when food webs are compared across habitats or time simple qualitative methods are used to conclude food-web shifts. However, more quantitative methods are either rarely used with the exception of [10], although they have been recently advocated [11–13], or methods do not exist.

Food-chain length (FCL) is one common metric used to describe food-web structure. Several hypotheses have been proposed to explain the variability in, and the factors determining, food-chain length [14,15]. Two such widely discussed, debated, and tested hypotheses are the dynamic constraints and the ecosystem size hypotheses [9]. The dynamic constraints hypothesis predicts that habitats subject to disturbance have shorter food chains because theoretical models predict longer food chains to be less resilient to disturbance or environmental variation and unattainable in nature [16]. However, there is only limited empirical evidence that supports the idea that dynamical stability limits food-chain length [9,17]. The ecosystem size hypothesis predicts food-chain length will be longer in larger ecosystems because of higher species diversity, habitat availability and heterogeneity [18], and more available resources [19]. These hypotheses all predict a linear relationship between FCL and ecosystem size or disturbance; consequently simple linear models have been used to test them, even though a more complex relationship may be driving FCL variability. To my knowledge, no study has simultaneously examined the relationship between natural variation in the physicochemical environment (multiple parameters) and changes in food-web structure through a multivariate approach.

In this study, environmental variables in combination with food-web metrics were used to track how and if the structure of food webs changes along a hydroperiod gradient. To do so, FCL and three community-wide metrics developed by Layman et al. [11]: δ^15^N range (represents extent of vertical structure), δ^13^C range (denotes niche diversification at base of food web), and total area (proxy for trophic diversity) were calculated from δ^13^C and δ^15^N values for nine ponds. A redundancy analysis (RDA), with community metrics and FCL as response variables and environmental variables as predictor variables was computed to estimate the environment’s influence on food-web properties. I predicted that ponds with a longer hydroperiod would be associated with larger size, higher species richness, and an increased FCL and δ^15^N range than ponds with a shorter hydroperiod. I also determined the relationship between invertebrate and amphibian presence and their physicochemical pond environments using Canonical Correspondence Analysis (CCA). The main objective was to extend the traditional single variable, linear regression approach of testing environmental influence on FCL to a multivariate analysis of key environmental predictors and food-web response variables and to relate this to community structure. If the ecosystem size and dynamic constraints hypotheses are upheld, then a strong association between area and FCL and hydroperiod and FCL should be found.

## Materials and Methods

### Study sites

I characterized aquatic food webs, amphibian and invertebrate communities, and examined their relationships with environmental variables for nine natural ponds varying in hydroperiod on the Queen’s University Biological Station (QUBS) property north of Kingston, Ontario, Canada (301km [187miles] Northeast of Toronto). The hydroperiod gradient spanned from intermittent freshwater woodland ponds to near-permanent freshwater marshes (classification of temporary waters follows Williams [2]). All of these ponds are naturally fishless; therefore, the top predators are expected to be predaceous invertebrates and/or salamander larvae.

### Field data collection

The hydroperiod varied between years in the study ponds. All ponds had water with partial ice coverage on the 3rd of April of both years. Link, Blue2, and QUBS ponds had drastically reduced hydroperiods in 2009 compared to 2008 (Table 1). For example, Blue2 dried a month earlier in 2009 (6 August) than in 2008 (between 20 September and 24 October). StowAway TidbiT temperature loggers (Onset Computer Corporation, Pocasset, MA, USA) were placed in the center of each pond at ~ 3cm from the bottom from 4 May until 24 Oct. 2008 and from 23 April until 29 Oct. 2009 or until the ponds dried (Table 1). Temperature was logged automatically every 6 hours in 2008 and every 5 hours in 2009. From this data water temperature values were reduced to mean daily water temperature per pond for the length of the pond hydroperiod. Monthly sampling of the physicochemical environment in 2008 included measuring pH, dissolved oxygen (mg/L), and air temperature (°C). Additionally, in 2009 I measured overstory density by Spherical Densiometer (Forestry Suppliers, Inc.), water depth (m), and pond area (m^2^). Water-quality measurements were taken by a hand-held Hydrolab Quanta multiparameter probe (Hach Environmental, Loveland, CO). To obtain percent canopy density, four canopy readings were made facing each cardinal direction counting the number of open squares on a spherical densitometer, the four readings were averaged, multiplied by 4.17, and the product subtracted from 100% (California Department of Pesticide Regulation, Environmental Monitoring Branch, SOP number: FSOT.002.01). We calculated the coefficient of variation (CV) to describe the dispersion of water temperature and water depth within a pond; the higher the CV, the greater the variability. Pond perimeter was mapped using a Trimble handheld GPS Pathfinder and pond area (m^2^) calculated in ArcMap.

**Table 1:** Hydroperiod (length of aquatic phase) and physical-chemical data for each pond.

Category	pond	Hydroperiod (# of days)		CV Temperature		Dissolved oxygen (mg/L)		pH		CV water depth	
		2008	2009	2008	2009	2008	2009	2008	2009	2008	2009
short	Winter1	65	74	0.271	0.31	6.38	6.68	7.38	7.14	NA	2.65
short	Winter2	65	74	0.243	0.21	2.65	6.35	7.04	6.82	NA	1.91
short	Snake	NA	102	NA	0.23	NA	3.61	NA	5.49	NA	1.06
intermediate	Link	147	126	0.205	0.30	1.21	5.17	7.31	6.90	NA	1.20
intermediate	Qubs	175	127	0.212	0.28	3.10	5.33	6.76	6.24	NA	0.85
intermediate	Blue2	175	125	0.302	0.19	7.11	5.80	7.73	7.18	NA	1.00
intermediate	Blue1	NA	140	NA	0.19	NA	5.48	NA	7.11	NA	0.94
long	Indian	365	365	0.28	0.39	5.04	6.23	7.27	6.47	NA	0.74
long	P82	365	365	0.24	0.34	4.79	6.62	7.42	6.84	NA	0.52
		**mean pond area (m^2^)**		**mean Perimeter: Area (m)**		**CV pond area**		**mean Canopy cover (%)**			
		2008	2009	2008	2009	2008	2009	2008	2009		
short	Winter1	NA	104.33	NA	0.83	NA	1.31	NA	50.26		
short	Winter2	NA	358.83	NA	0.91	NA	1.35	NA	75.91		
short	Snake	NA	190	NA	0.44	NA	0.95	NA	64.62		
intermediate	Link	NA	188	NA	0.71	NA	1.19	NA	88.36		
intermediate	Qubs	NA	505.03	NA	0.93	NA	1.21	NA	47.58		
intermediate	Blue2	NA	844.5	NA	0.24	NA	0.83	NA	28.02		
intermediate	Blue1	NA	1258.15	NA	0.27	NA	0.8	NA	8.03		
long	Indian	NA	3488.84	NA	0.09	NA	0.22	NA	3.96		
long	P82	NA	190.35	NA	0.38	NA	0.61	NA	25.54		

Ponds Snake and Blue1 were not sampled in 2008. CV is the coefficient of variation. NA means data was not collected.

We collected invertebrates and amphibians by dip-netting from numerous areas within each pond every two weeks (April—September in 2008 and 2009) to comprehensively sample multiple trophic pathways leading to top consumers. Because of the relatively low species richness (four to nine species per pond) we could sample the entire amphibian community. I sorted major invertebrate groups in the field, placing samples in plastic jars, and holding them on ice until deposited in a freezer. Amphibians were euthanized by immersion in Tricaine Methanesulfonate (MS-222) buffered with an equal amount of sodium bicarbonate to a pH of ~7.0 in accordance with animal care guidelines approved by the University of Toronto and Queen's University Animal Care Committees (# 20007692), held on ice during field collection, and then frozen until further processing. Animals were collected under scientific collector’s permit (# 1051193) approved by the Ontario MNR.

Food-web resources were collected monthly from each pond for isotopic analysis during the spring, summer, and fall seasons (April to September) of 2008 and 2009. Basal organic resources consisted of detritus, aquatic macrophytes (submerged and emergent), algae, seston, and fine benthic organic matter (FBOM). Seston was acquired by collecting a 1000 mL water sample at mid-depth in the water column from each pond. FBOM was sampled by dredging a 53μm net across the bottom of the pond in several random locations. The net contents were emptied onto a sieve tower of decreasing mesh size. The benthic matter collected on the 53 μm sieve was rinsed with distilled water into a collection jar. Samples were held on ice during field collection and then frozen until processed.

### Laboratory work and stable isotope analysis

Seston containing zooplankton, bacteria, and phytoplankton were obtained by filtering water samples onto pre-combusted Whatman glass microfiber GF/F filters. FBOM was poured into glass scintillation vials, allowed to settle, and the clear liquid was suctioned off using a pipette and discarded. The resultant concentrated FBOM was dried in scintillation vials. Aquatic macrophytes, algae, and coarse detritus samples were rinsed of attached periphyton or sediment and invertebrates (removal checked under a dissecting microscope). Tadpoles and salamander larvae were identified to species using specialist keys [20–22]. Using Merritt & Cummins [23] and Marshall [24] as guides, I identified and counted invertebrates to family or genus level under a dissecting microscope. Whole specimens of invertebrates (snails and clams without shells), tadpoles (minus guts) and salamander larvae (minus stomachs) were used for stable isotope analysis (SIA). Replicates of amphibians represent single individuals, whereas replicates of invertebrates were composite samples of 2 to 50 individuals. All amphibian, invertebrate, and, basal resource samples were rinsed with distilled water, dried in a drying oven at ~ 60°C for 2–3 days, and ground to a homogeneous powder in a Mini-Beadbeater (BioSpec products Inc., OK, USA) for use in SIA. All samples were prepared in the Williams lab at the University of Toronto Scarborough and shipped to the Cornell Isotope Laboratory (COIL) for isotopic analysis. Stable isotope values are reported in delta notation as δ^13^C or δ^15^N using the equation δ^13^C or δ^15^N = ((R_sample_/R_standard_)– 1) × 1000, where R is ^13^C: ^12^C or ^15^N: ^14^N. The standard is Vienna Pee Dee Belemnite for δ^13^C and atmospheric nitrogen for δ^15^N. Mathematical standardization was used to account for lipid variation in δ^13^C in consumer samples (mean amphibian: 2008 C:N = 4.2, 2009 = 3.8; mean invertebrate: 2008 C:N = 5.0, 2009 = 4.9) using the recommended equations from [25]. We used δ^15^N because it predictably increases through trophic transfer up the food chain, providing an estimate of vertical trophic position [26,27]. δ^13^C was used to estimate assimilation of basal energy sources by consumers [28,29], because different energy sources often show distinct δ^13^C signatures with little fractionation up the food chain [30].

Realized food-chain length was estimated as the maximum trophic position obtained by a species from each pond [31]. Trophic position was calculated for each individual using the site mean δ^15^N of basal resources as a baseline (S2 Table), as done in Winemiller et al. [32].

$$TP_{SI}=\lambda +\left(\delta^{15}N_{sc}-\delta^{15}N_{baseline}\right)/\Delta n$$

where λ is the trophic level of the baseline (1 for basal resources), δ^15^N_sc_ is the nitrogen isotope signature of the consumer being evaluated and δ^15^N_baseline_ is the mean nitrogen isotope signature of basal resources (algae, detritus, seston, FBOM, and aquatic plants). I used 2.3‰ based on [33] for aquatic animals for Δn, which is the trophic level enrichment in δ^15^N value. Recent reviews of consumer-diet δ^15^N enrichment [33–36] do not provide estimates for amphibians. Vanderklift and Ponsard [35] found that organisms consuming detritus had significantly lower estimates of Δ and that most aquatic organisms are ammonolectic, which averaged 2.0‰ per trophic step. Many researchers use Δ of 3.4‰ based on Minagawa and Wada [37] and Post [31]. I decided on the 2.3‰ value because it is specific to aquatic animals and is an intermediate value across the range of available estimates.

The community-wide metrics, δ^15^N range, δ^13^C range, and total area encompassed by the consumer food web (TAfoodweb) were calculated for each pond using mean consumer species δ^15^N and δ^13^C values. Total area was calculated using the geometry [38] package in R [39] as the convex hull area encompassed by the pond community in isotopic space. The total area metric as proposed by Layman et al. [11] has been criticized by Hoeinghaus and Zeug [40] because they did not standardize isotopic values. According to Cornwell et al. [41] it is not appropriate to calculate convex hull measurements on non-transformed data because traits are measured in different units and often have different variances, which is true for δ^15^N and δ^13^C. Therefore, we log transformed δ^15^N and δ^13^C values for the convex hull calculations. No amphibians were found in Winter1 during the 2009 collection period; therefore TAfoodweb calculated the extent of invertebrate trophic diversity, whereas other TAfoodweb calculations included invertebrates and amphibians. Layman et al. [11] states the convex hull of the consumer food web approximates the total area of the food web. I think this metric is a better descriptor of the community trophic space because the metric does not use the resources available to consumers in the food web therefore, it is not estimating the entire food-web space.

### Statistical analyses

Amphibian species and invertebrate family data sets were analyzed separately using correspondence analysis (CA) on presence-absence data. These communities were compared with the pond environmental data sets using canonical correspondence analysis (CCA). This direct gradient multivariate method summarizes the maximum amount of variation in the community data set while constraining it to axes associated with the environmental data [42].

A redundancy analysis (RDA) was performed to test the influence of environmental factors on food web components. RDA is a direct gradient ordination method that can be used to test if species composition is related to a set of measured variables. RDA is similar to principal components analysis because the Euclidean distances among the objects in ordination space are preserved [43]. In the RDA ordination plot, the axes represent linear combinations of the explanatory variables. The length of the arrow relates to the strength of the relationship with the axis. RDAs were performed separately for 2008 and 2009 using means (or otherwise stated) of each predictor and response variable computed from the entire data set (April to October). The environmental predictor or constraining variables were: mean pH, mean dissolved oxygen (mg/L), hydroperiod (number of days with water), and the coefficient of variation (CV) of water temperature (°C). The response variables were food-chain length (maxTP), δ^15^N range, δ^13^C range, and total area encompassed by the community trophic space (TAfoodweb) for 2008 and 2009 analyses. An extra RDA was run for 2009 data using three additional environmental variables that were not measured in 2008 (i.e., overstory density (meanCanopy), CV of water depth (m; CVdepth), and mean pond area (m^2^; meanArea). The variance inflation factor (VIF), a statistic to detect multicollinearity among independent variables, was calculated for all predictor variables for each year. VIF values were low (≤ 5), unless otherwise stated, so all variables were kept in further analyses. By using this approach, we show which ponds are characterized by particular environmental variables and whether food webs from a particular pond or group of ponds could be attributed to specific measured community metrics [44]. I conducted permutation tests on the CCA and RDA models to test the significance of constraints (environmental variables). Multivariate statistics were computed in R version 2.15 using the vegan package [45]. Summary statistics were calculated using MYSYSTAT 12 [46].

## Results

### Food web-environment relationships

**Fig 1:**
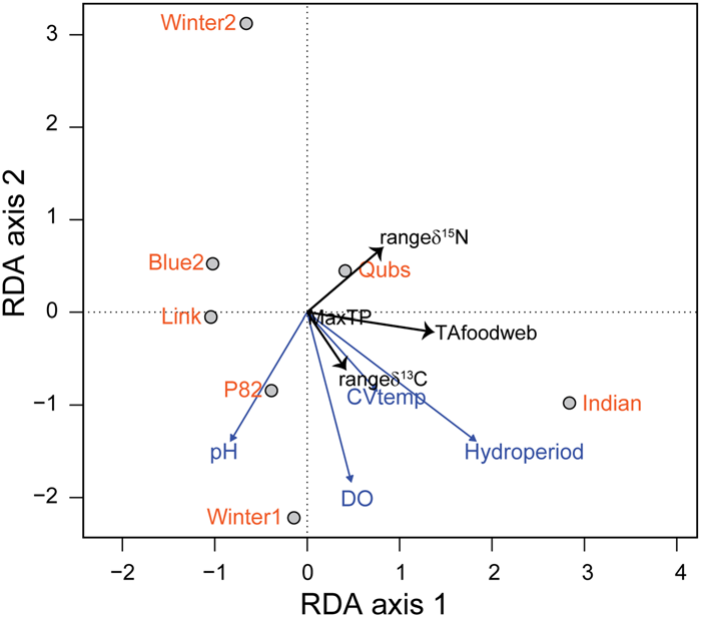
Ordination plot for 2008 showing variation in food-web structure (n = 4 variables, black arrows) in response to environmental variation (n = 4 variables, grey arrows) summarized by redundancy analysis (RDA). Sampling sites are denoted by black outlined grey circles. MaxTP is the trophic position of the top predator or realized food-chain length, TAfoodweb is the total area encompassed by all species in δ^13^C–δ^15^N bi-plot space. Plot used scaling = 1 to create a distance biplot where objects approximate their Euclidean distances in the space of response variables. Length of the arrow represents the strength of the gradient. Arrows that are directed in opposite directions are negatively correlated. The angles between environmental and food-web variables reflect their correlations.

**Fig 2:**
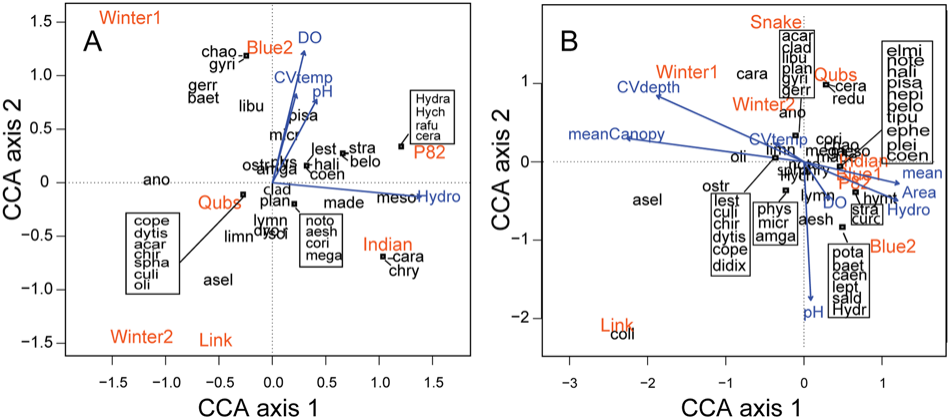
Canonical correspondence analysis (CCA) ordination biplots showing the association between the presence-absence of invertebrate families and environmental predictors in (A) 2008 and (B) 2009. (A) Four environmental predictors in 2008 measured in seven ponds and (B) seven environmental variables from nine ponds in 2009. The direction of the arrow indicates direction of maximum change and the length is proportional to the rate of change of that variable. See S1 Table for full invertebrate family names.

**Fig 3:**
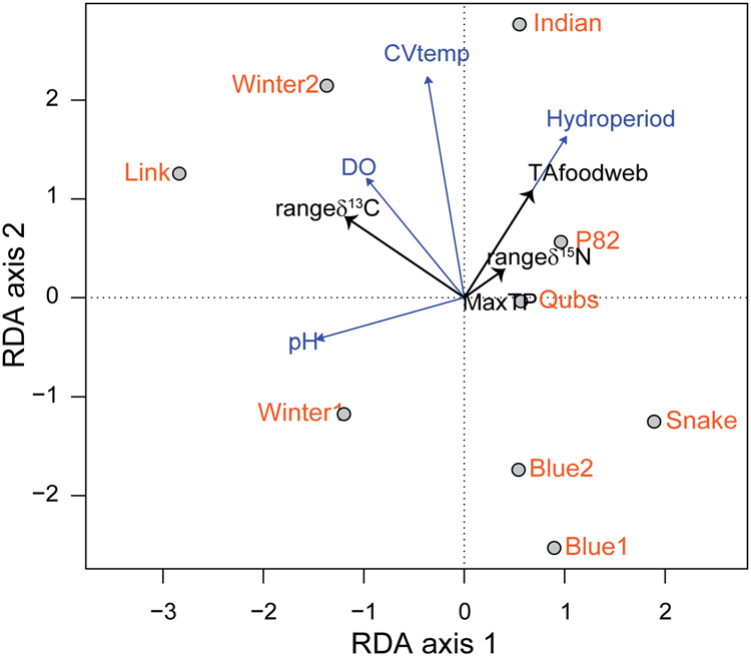
Redundancy Analysis (RDA) tri-plot illustrating the relationship between environmental variables (n = 4, grey arrows), food-web metrics (n = 4, black arrows), and ponds (n = 9, black outlined grey dots) in 2009. Pond name is placed adjacent to dot.

Variation in food-web structure in response to changing environmental conditions was observed between sampling years. In both years the RDA models indicated that high amounts of variation in food-web structure could be explained by the environmental predictors (68% and 52%, respectively). Two important commonalities between years are that trophic diversity (TAfoodweb) positively covaried with hydroperiod and that FCL (MaxTP) was correlated to multiple environmental and food-web metrics. The largest pond (Indian pond) with the longest hydroperiod had relatively the same association with environmental variables (e.g., high temperature variability, high DO) and food-web structure (high trophic diversity and range in δ^13^C) between years. Generally ponds with a longer hydroperiod were associated with higher trophic diversity, higher species richness (Figs 1, 2 and 3), and an increased δ^15^N range (Fig 3) than ponds with a shorter hydroperiod.

In the 2008 RDA, the first axis (RDA axis 1) explained 78% of the variability and described a gradient with ponds ranging from short hydroperiod, higher pH, low temperature variability, and low dissolved oxygen (negative side of axis 1) to longer hydroperiod ponds with high variation in temperature, high dissolved oxygen, and high trophic diversity (TAfoodweb) (Fig 1). Trophic diversity was strongly influenced by hydroperiod which is validated by the greater number of invertebrate taxa on the right half of the CCA ordination (Fig 2A). RDA axis 2 explained 21% of the variation in the food web-environment relationship where the range in δ^15^N opposed all environmental variables and other food-web metrics were related in the same direction. The permutation test based on all constrained eigenvalues was not significant (*P* = 0.45). Winter2 was distinct from other ponds in terms of food-web structure and response to environmental variables. MaxTP (FCL) was weakly related to Axis1 and 2, therefore positioned near the origin of the ordination plot, indicating either: 1) an overall general response to all variables rather than strongly responding to a single environmental variable; or 2) a low response to all environmental variables. MaxTP was strongly positively correlated with hydroperiod (r = 0.74) and the total area of the food web (TAfoodweb; r = 0.91) which suggests the longer the hydroperiod the longer the food-chain length; with a longer food chain conferring a larger food-web area and higher trophic diversity. However, MaxTP was correlated to the other food-web metrics (range δ^13^C r = 0.78, range δ^15^N r = 0.44) causing the variable to locate at the origin of the ordination.

The first axis (RDA axis 1) of the corresponding RDA model using 2009 community-wide metrics and the four environmental variables explained 59% of the variation and was mostly summarized by pH and dissolved oxygen in one direction and hydroperiod in the other. The range δ^13^C was negatively related to hydroperiod, suggesting higher resource diversity in shorter hydroperiod ponds (Fig 3). The second axis (RDA axis 2) explained 40% of the variation and was most strongly associated with variation in water temperature (CVtemp). The range in δ^13^C was positively correlated with dissolved oxygen. TAfoodweb and range δ^15^N responded positively to hydroperiod, meaning community trophic space and the vertical extent of the food web increased with longer hydroperiod. Short and intermediate hydroperiod ponds had less temperature variation with the exception of Winter2. Longer hydroperiod ponds (QUBS, Indian, and P82, especially) had strong associations with the range of δ^15^N and trophic diversity (TAfoodweb), but weaker association with food-chain length (MaxTP). MaxTP was again weakly associated with axis 1 and 2. The permutation test based all constrained eigenvalues was not significant (*P* = 0.4).

**Fig 4:**
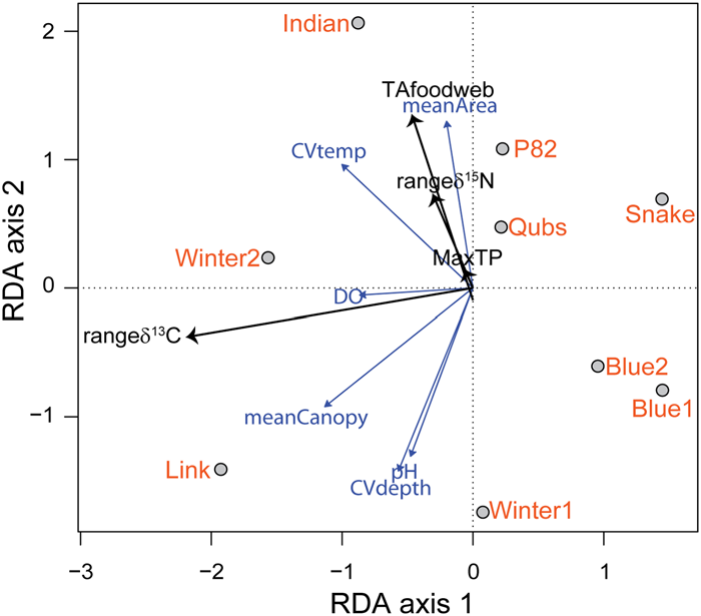
RDA tri-plot displaying food-web structure (black arrows) in relation to six environmental variables (grey arrows, excluding hydroperiod) and nine ponds (black outlined grey dots) in 2009.

The RDA model on the expanded 2009 environmental data set (7 variables) explained 99% of the variation leaving only 1% unexplained by the environmental variables in the model, meaning the model was very close to over fitting the data. Examination of the VIF indicated hydroperiod was highly correlated to other variables. Given this, hydroperiod was removed from the dataset and the RDA was performed again using six environmental variables. The conclusions were similar between the analyses (with and without hydroperiod) because of the similar number of environmental variables to the number of sites. In 2009, 97% of the variation in food web structure was explained by the environmental variables in the expanded RDA model, indicating that food web variables could be explained mostly by the environmental variables even without hydroperiod in the model. All constrained canonical axes significantly summarized relationships between the food-web metrics and the environmental variables (permutation test *P* = 0.01). The first axis explained 66% of the variation and approximated a gradient from ponds characterized by higher dissolved oxygen and temperature variability, dense canopy cover, and a broader range in δ^13^C (left side of origin) most closely associated with Link (mean canopy cover = 88%) and Winter2 ponds (mean DO = 6.35 mg/L) (Fig 4). The second axis (RDA axis 2) summarized 27% of the variation and was polarized on one end by pond area and CV water temperature (upper half of ordination plot) and pH, CV water depth, and canopy cover on the other end (lower half of ordination plot). Indian pond was characterized by a large pond size, moderate variation in water temperature, higher dissolved oxygen, and a food web with increased δ^15^N range and trophic diversity. In contrast, Winter1 was the smallest pond with the shortest food-chain length (MaxTP), narrower δ^13^C and δ^15^N ranges, and far less trophic diversity than Indian pond. Food-chain length (MaxTP) was positioned near the center of the ordination at the origin of the environmental vectors (Fig 4).

### Insect and amphibian community-environment relationships

The CA of the invertebrate community in 2008 explained 49% and in 2009 46% of the total variance in each of the first two axes (Table 2). Ordination plots of invertebrate families contrast drought-tolerant families with short and intermediate hydroperiod ponds on the left side of axis 1 and less drought-resistant families with long hydroperiod ponds to the right of the axis (S1 and S3 Figs).

**Table 2:** Results from Correspondence Analysis (CA) on the invertebrate and amphibian assemblages (presence-absence data).

Year	Amphibian assemblage				Invertebrate assemblage			
		Eigenvalue				Eigenvalue		
	CA total inertia	Axis 1	Axis 2	Variation explained by axes 1 and 2 (%)	CA total inertia	Axis 1	Axis 2	Variation explained by axes 1 and 2 (%)
2008	0.42	0.222	0.098	75	0.67	0.191	0.142	49
2009	0.61	0.255	0.190	73	0.80	.220	0.145	46

CA reports total Inertia, which is a multivariate measure of the amount of variation in data set.

In 2008, CCA results estimated 70% of the total variation in the invertebrate community was explained by the environmental variables (Table 3). Axis 1 represented a gradient of short hydroperiod ponds and low family richness (far left side of the ordination) to more taxon rich and longer hydroperiod ponds (far right, Fig 2A). The two shortest hydroperiod ponds (Winter1 and Winter2) were most closely associated with Anostraca (fairy shrimp, a temporary pond specialist), intermediate hydroperiod ponds associated with numerous taxa from beetles (Dytiscidae, Halipidae), dragonflies and damselflies (Libellulidae, Lestidae, Coenagrionidae), and caddisflies (Limnephilidae) to snails (Physidae, Lymnaeidae, Planorbidae) and clams (Sphaeriidae), whereas the two longest hydroperiod ponds were associated with Diptera (Ceratopogonidae), true bugs (Mesoveliidae, Nepidae), and beetles (Hydrophilidae, Hydraenidae) (Fig 2A). CCA axis 2 summarized variation in DO, CV of temperature, and pH, which were positively correlated. Blue2 was characterized by high dissolved oxygen, Gyrinidae beetles and Chaoboridae Phantom Midge larvae which were polarized by Link pond exhibiting low dissolved oxygen, little variation in temperature, low pH and most closely associated with isopods (Asellidae) that are tolerant of low dissolved oxygen. The permutation test for significance based on the CCA constrained by four environmental variables was not significant (*P* = 0.15).

**Table 3:** Eigenvalues, percentage of variation explained, and permutation test statistics from Canonical Correspondence Analysis (CCA) of invertebrate and amphibian assemblages and environmental data from ponds in Southeastern Ontario, Canada.

		Eigenvalue					
	Total variation explained by environment (%)	Axis 1	Axis 2	Variation explained by axes 1 and 2 (%)	No. environmental variables	Pseudo-F all axes	*P*-value all axes
Amphibian assemblage							
2008	53	0.094	0.082	79	4	0.561	0.84
2009	49	0.25	0.17	77	5	1.768	0.17
Invertebrate assemblage							
2008	70	0.187	0.124	66	4	1.161	0.15
2009	75	.202	0.129	52.5	7	1.008	0.40

Using CCA, 87% of the total variation in the 2009 invertebrate community was explained by the environmental data set (Table 3). In agreement with the CA, all short hydroperiod ponds and one intermediate hydroperiod pond fell on the left of axis 1. CCA axis 1 described a gradient from shorter hydroperiod, smaller ponds with dense canopy cover, high variation in water depth and temperature (left of origin in ordination) to longer hydroperiod, larger ponds with more open canopies and less variation in water depth and water temperature (right of origin; Fig 2B). The second CCA axis was strongly determined by pH. Therefore, there were three main factors controlling aquatic invertebrate communities in 2009, one is related to physical pond properties, pond area and hydroperiod, the second and negatively correlated to the first is related to intrinsic pond conditions, variation in water depth and temperature, and extrinsic conditions (canopy cover) which can influence the intrinsic conditions. The third factor was orthogonal, therefore unrelated to the other two factors, and was influenced by water chemistry, pH and DO. Blue2 and Blue1 (intermediate hydroperiods) were opposite of Winter1 and Winter2 (short hydroperiods) showing that these pond pairs contained different invertebrate fauna. Blue2 was characterized by high dissolved oxygen and accordingly the only pond associated with Ephemeroptera taxa (Potamanthidae, Baetidae, Caenidae; Fig 2B). The permutation test for significance based on the 2009 CCA was not significant (*P* = 0.5).

**Fig 5:**
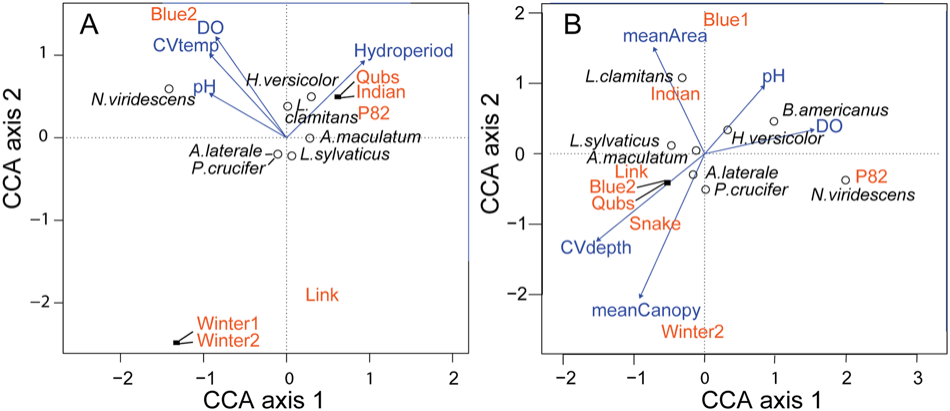
Canonical correspondence analysis (CCA) biplots showing the association of amphibian species presence-absence and A) four environmental predictors in 2008 from seven ponds and B) five environmental variables in 2009 collected from eight ponds. Species placement in biplot is marked with an open circle. MeanCanopy is the mean canopy cover, meanArea is the mean pond area (m^2^) measured over the ponds duration, CVdepth is the coefficient of variation of water depth, DO is dissolved oxygen, and CVtemp is the coefficient of variation of water temperature.

Patterns of amphibian occurrence and environmental conditions across ponds can be seen between years. The CA of the amphibian communities in 2008 and 2009 summarized similar amounts of variance (Table 2). The shortest hydroperiod ponds (Winter1 and Winter2) were most similar to each other and most different from longer hydroperiod ponds, including QUBS, Indian, and P82, which had higher species richness and different amphibian composition. Most species were positioned near the center of the ordinations because they are associated with more than one pond and species co-occur in ponds (Fig 5 and S4 Fig). Ponds that harbored *Notophthalmus viridescens* (red-spotted newt) had distinct amphibian assemblages (S2 and S4 Figs) and were often associated with higher DO, temperature variation, and pH (Fig 5).

The CCA of amphibian species and environmental data in 2008 explained 53% of the total variability in the amphibian community (Table 3). The first axis (CCA1) was mostly strongly influenced by hydroperiod, meanwhile the other variables had similar influence but of opposite direction on the axis (Fig 5A). Dissolved oxygen (DO) was strongly associated with the second axis (CCA2) as was CV of temperature, hydroperiod, and to a lesser degree pH. *Ambystoma maculatum* (spotted salamander) was positioned in the origin because it was found in all ponds except those with the two shortest hydroperiods. The permutation test for significance based on the CCA was not significant (*P* = 0.84) which was constrained by four environmental variables.

No amphibians were found in Winter1 during 2009, so it was not included in the CA and CCA analyses. Using all seven environmental variables in the CCA caused the model to overfit the data. Examination of the VIF values indicated hydroperiod and CV water temperature were highly correlated with the other variables. Given this, we removed these variables from the dataset and re-ran the CCA using the remaining five environmental variables. The CCA of amphibian species and environmental data in 2009 explained approximately 82% of the total variation in the amphibian community. Most of the variation on CCA Axis 1 was associated with two opposing responses, varitaion in water depth and dissolved oxygen (Fig 5B, Table 3). High dissolved oxygen was positively correlated with pH and associated with *Bufo americanus* and *Hyla versicolor*. In contrast, low dissolved oxygen, lower pH and high variation in water depth described pond communities containing *Ambystoma maculatum* and *Lithobates sylvaticus* (left side of ordination; Fig 5B). The second axis explained the variation in species presence-absence in ponds by a strong negative relationship between pond area and canopy cover (proportion explained was 32%). Winter2 was a small pond with dense canopy cover harboring *Psuedacris crucifer* and *A*. *laterale* (lower half of ordination), whereas *Lithobates clamitans* was found only in larger ponds with open canopies, most often in Indian pond. The permutation test for significance based on the CCA constrained by five environmental variables was not significant (*P* = 0.17).

## Discussion

Invertebrate and amphibian communities were structured by the pond hydroperiod which in turn, especially for the invertebrates, influenced the trophic diversity of the food web. Trophic diversity responded strongly to hydroperiod, pond area, and variation in water temperature in both years. Larger ponds with longer hydroperiods conferred higher species richness and trophic diversity. This was best illustrated in 2009 when invertebrate richness ranged from 17 to 41, and amphibian species richness ranged from 3 to 6 from short to longer hydroperiod ponds. The range in δ^13^C also responded strongly to hydroperiod and variation in temperature in 2008, but was influenced by dissolved oxygen, temperature variation, pH, and canopy cover in 2009. Higher δ^13^C range was seen in Indian pond in 2008, this is a large pond with a long hydroperiod, mostly open canopy, emergent and non-emergent aquatic plants, a large detrital component from leaf fall and aquatic plant senescence, and a rich invertebrate community. The range in δ^13^C was positively correlated with dissolved oxygen indicating that ponds such as Link, which had high dissolved oxygen, also support diverse basal production sources (range δ^13^C). Range in δ^13^C was often positively correlated with total area of food web, indicating a strong association and a similar response to environmental variation. A wide δ^13^C range suggests organisms in the community have the opportunity to feed on a diverse set of resources [11]. A greater diversity of resources could possibly allow for higher trophic diversity and longer food chains [18].

The results presented clearly show that FCL (MaxTP) was influenced by multiple environmental variables and was not strongly responsive to any one particular variable thereby forcing it to the center of the ordinations. This finding that multiple controls may influence FCL has previously been acknowledged [9], but most studies still test the univariate relationship between an environmental variable and FCL (see studies on ecosystem size [18,47–49] and environmental disturbance and/or variation [49–51]). Therefore, my results do not directly support the ecosystem size or dynamic constraints hypotheses. Instead they show a trend that both controls are important in concert with the physical-chemical conditions of the habitat. Predictors of environmental variation, in particular temperature and depth, were associated with other measures of food-web structure (variation in δ^15^N, δ^13^C, and trophic diversity). Longer FCLs are attributed to larger habitat size through mechanisms of higher species richness, habitat heterogeneity [9,52], or changes in the degree of trophic complexity or omnivory [53] which is most often seen in lakes or habitats bearing fish. In this study, trophic diversity and in a previous study, species richness [49] positively correlated with pond area; however, FCL (MaxTP) was only weakly correlated. These results give partial support for the productive-space hypothesis which states that ecosystem size allows for a greater FCL through an increase in food availability [19]. In a recent meta-analysis, Takimoto and Post [17] found significant positive mean effects of size and productivity indicating support for the ecosystem size hypothesis and agreed that the productive-space mechanism could shape the FCL-ecosystem size relationship. In summary, other aspects of food-web structure besides FCL (i.e., trophic diversity, range in δ^15^N and δ^13^C) are influenced by environmental variation in temperate ponds.

There are a few potential reasons for lack of support for the ecosystem size and dynamic constraints hypotheses. Community-wide metrics of food-web structure correlated weakly to moderately with FCL, which could decrease the reliability of predicting the relationship between FCL and other environmental predictors. Alternatively, perhaps the environmental gradients among ponds were not large enough to elicit a change in FCL or small sample size. However, the study ponds ranged in size from 78 m^2^ to almost 3500 m^2^ and differed in hydroperiod by 291 days between the shortest and longest hydroperiod pond. Additionally, despite disparity in environmental conditions the presence of the top predator of the system (usually *A*. *laterale* or *A*. *maculatum*) was consistent across ponds. Therefore, similarity in the top predator species could have constrained food-chain length amongst the ponds preventing large differences among the habitats under study. One must also take into consideration the majority of studies to compare our results against are from lakes and streams. Woodland ponds are an ideal system to evaluate the mechanisms driving variation in food-web structure; however they differ from larger aquatic ecosystems such as lakes in a few key ways. For instance, in comparison to lakes, ponds are generally much smaller and often fishless, they show fluctuating colonization and migration dynamics changing daily and seasonally, pronounced environmental variation, and different allochthonous resource base supporting the food web. Cross-habitat studies are essential to recognize patterns of food-web structure.

A consistent pattern was seen when pond communities were compared across years. Variation in water temperature and dissolved oxygen were positively correlated and associated with the variation in δ^13^C. *Notophthalmus viridescens*, Veliidae, Ephemeroptera and Trichoptera taxa were more common in ponds having higher dissolved oxygen. On the other hand, ponds that had an intermediate hydroperiod (Blue1, Blue2, QUBS, Link) showed some similarities in associations with certain environmental variables, most often low temperature variation, dissolved oxygen, and pH, but did not show consistent patterns for food-web variables across years. This result exemplifies the strong environmental variability of intermittent ponds. Magnusson and Williams [54] found natural temporal variation to be more important than biological factors in shaping the physicochemical environment in four fishless, intermittent ponds. Therefore, these results suggest a common feature of intermittent systems in that they share strong associations among environmental variables making it more difficult to elucidate one mechanism driving food-web structure.

## Conclusions

Most food-web studies test the effect of one environmental variable on FCL. By extending the effects on FCL to a multivariate approach, I was able to evaluate the relative influence of several abiotic variables on multiple food-web components including FCL. I found short hydroperiod ponds are characteristically different in their amphibian and invertebrate communities and differ markedly from longer hydroperiod ponds in terms of food-web responses and environmental predictors. I found the determinants of food-chain length in relatively small, isolated wetlands differed from the determinants in other habitat types (e.g., ecosystem size is a strong determinant of food-chain length in lakes). This reinforces the need for further investigation of the structure and function of temporary water systems. Small ponds are exceptionally useful to study because the scale of the system is directly relevant to the scale of the mechanisms driving ecosystem processes. In conclusion, I argue that to better understand diverse environmental influences on food-web dynamics, a multivariate approach including interacting factors should be adopted in theoretical and empirical research.

## Supporting Information

S1 Fig Correspondence Analysis (CA) comparing invertebrate assemblage structure (presence-absence data) among ponds in 2008. (DOCX)

S2 Fig Correspondence Analysis (CA) comparing amphibian species assemblage structure (presence-absence data) among ponds in 2008. (DOCX)

S3 Fig Correspondence Analysis (CA) comparing invertebrate assemblage structure (presence-absence data) among ponds in 2009. (DOCX)

S4 Fig Correspondence Analysis (CA) of amphibian species assemblage structure (presence-absence data) among ponds in 2009. (DOCX)

S1 Table Abbreviations used in multivariate analyses, CA and CCA, for invertebrate families collected from nine woodland ponds in Ontario, Canada. (DOCX)

S2 Table Stable isotope baseline data used to calculate trophic position. (DOCX)
