# Supplementary material for: Food Webs in Relation to Variation in the Environment and Species Assemblage: A Multivariate Approach
Source: PLoS One. 2015 Apr 16;10(4):e0122719. doi: 10.1371/journal.pone.0122719 (PMC4399920; doi:10.1371/journal.pone.0122719)
Supplement: S3 Fig — (DOCX) [file pone.0122719.s003.docx]

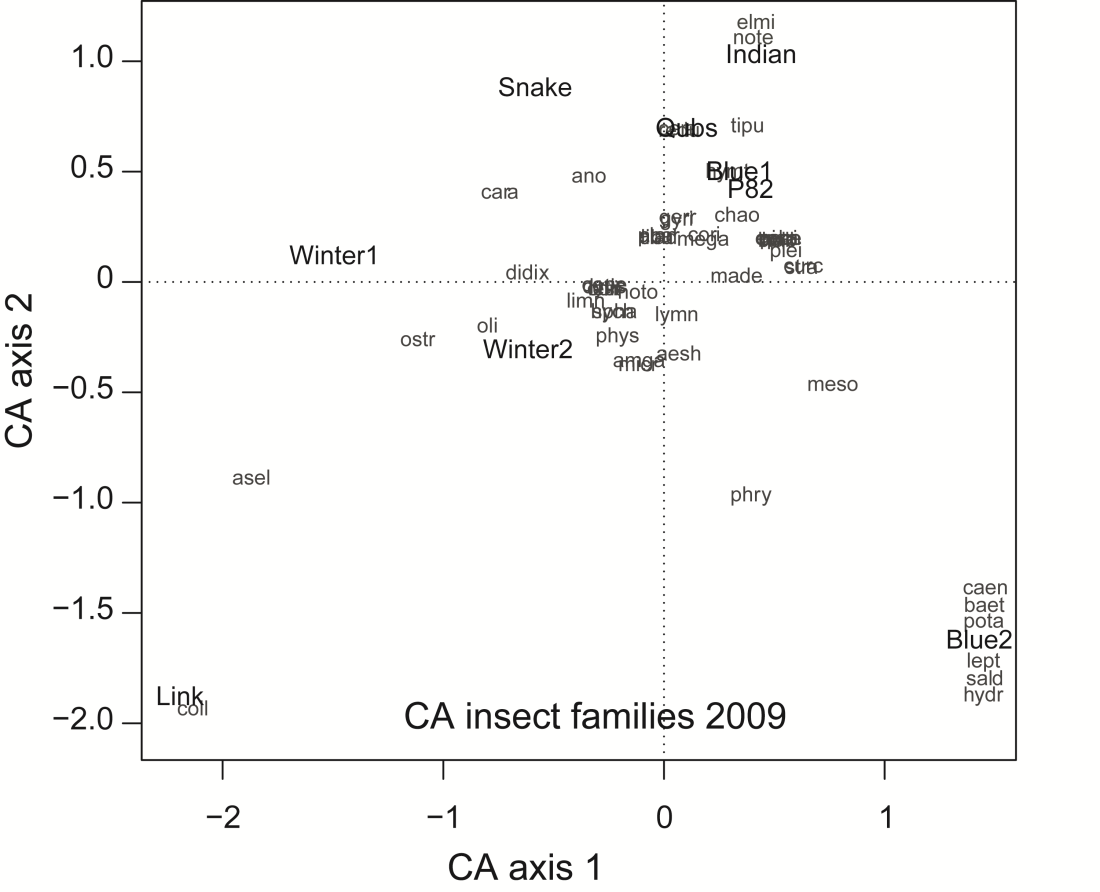


S3 Figure. Correspondence Analysis (CA) comparing invertebrate assemblage structure (presence-absence data) among ponds in 2009. Some species are stacked at the same location in ordination space.
