# Supplementary material for: Food Webs in Relation to Variation in the Environment and Species Assemblage: A Multivariate Approach
Source: PLoS One. 2015 Apr 16;10(4):e0122719. doi: 10.1371/journal.pone.0122719 (PMC4399920; doi:10.1371/journal.pone.0122719)
Supplement: S4 Fig — (DOCX) [file pone.0122719.s004.docx]

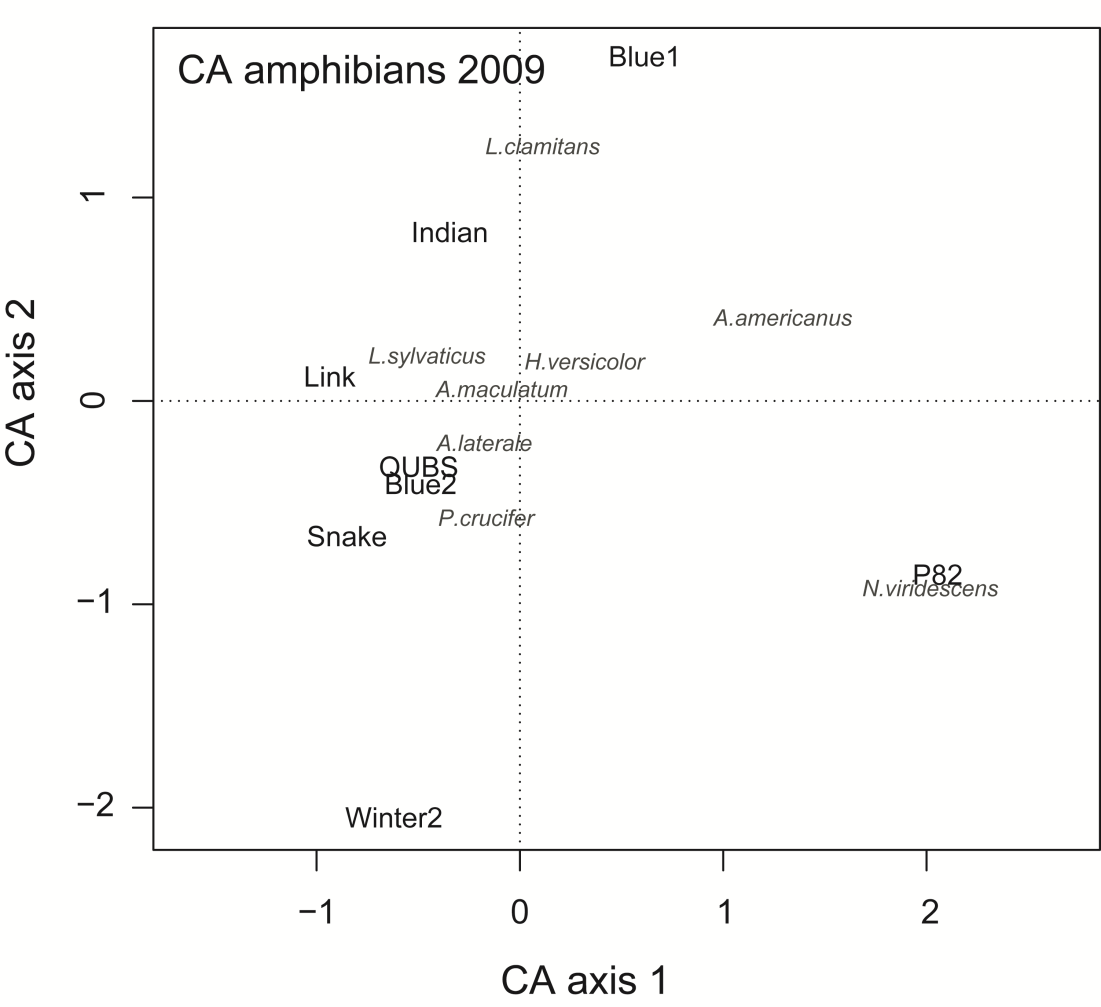


S4 Figure. Correspondence Analysis (CA) of amphibian species assemblage structure (presence-absence data) among ponds in 2009.
