# Supplementary material for: Food Webs in Relation to Variation in the Environment and Species Assemblage: A Multivariate Approach
Source: PLoS One. 2015 Apr 16;10(4):e0122719. doi: 10.1371/journal.pone.0122719 (PMC4399920; doi:10.1371/journal.pone.0122719)
Supplement: S1 Table — (DOCX) [file pone.0122719.s005.docx]

S1 Table Supporting Information

S1 Table. Abbreviations used for invertebrate families in multivariate analyses, CA and CCA. Invertebrates collected from nine woodland ponds in Ontario, Canada in 2008 and 2009. Presence (collected) indicated by 1, absence (not collected/found) indicated by 0. NA represents taxa that were not collected in one year of sampling but were found in another year. Pond abbreviations in table are as follows for short hydroperiod pond: W1 = winter1, W2 = winter2, Sn = snake, intermediate hydroperiod: Li = link, Qu = Qubs, B2 = Blue2, B1 = Blue1, and long hydroperiod: In = Indian, P = P82.

|  |  | 2008 | | | | | | |  | 2009 | | | | | | | | |
| --- | --- | --- | --- | --- | --- | --- | --- | --- | --- | --- | --- | --- | --- | --- | --- | --- | --- | --- |
|  |  | Hydroperiod | | | | | | |  | Hydroperiod | | | | | | | | |
|  |  | short | | intermediate | | | long | |  | short | | | intermediate | | | | long | |
| Order (sublevel), family | 2008 code | W1 | W2 | Li | Qu | B2 | In | P | 2009 code | W1 | W2 | Sn | Li | Qu | B2 | B1 | In | P |
| (Acari; Hydracarina) | acar | 1 | 1 | 1 | 1 | 1 | 1 | 1 | acar | 1 | 1 | 1 | 0 | 1 | 1 | 1 | 1 | 1 |
| (Clitellata; Hirudinea) | made | 0 | 0 | 0 | 1 | 0 | 1 | 1 | made | 0 | 1 | 0 | 0 | 1 | 1 | 0 | 1 | 1 |
| (Clitellata; Oligochaeta) | oli | 1 | 1 | 1 | 1 | 1 | 1 | 1 | oli | 1 | 1 | 0 | 1 | 1 | 0 | 0 | 0 | 1 |
| Amphipoda | amga | 1 | 0 | 1 | 1 | 1 | 1 | 1 | amga | 0 | 1 | 0 | 1 | 1 | 1 | 1 | 0 | 1 |
| Anostraca | ano | 1 | 1 | 0 | 1 | 0 | 0 | 0 | ano | 1 | 1 | 1 | 0 | 1 | 0 | 1 | 1 | 0 |
| Araneae | pisa | 1 | 0 | 0 | 0 | 1 | 1 | 1 | pisa | 0 | 0 | 0 | 0 | 1 | 1 | 1 | 1 | 1 |
| Basommatophora, Lymnaeidae | lymn | 0 | 1 | 1 | 1 | 1 | 1 | 1 | lymn | 0 | 0 | 0 | 1 | 1 | 1 | 1 | 1 | 1 |
| Basommatophora, Physidae | phys | 1 | 0 | 1 | 0 | 1 | 1 | 1 | phys | 1 | 0 | 0 | 1 | 0 | 1 | 1 | 1 | 1 |
| Basommatophora, Planorbidae | plan | 0 | 1 | 0 | 1 | 1 | 1 | 1 | plan | 1 | 1 | 1 | 0 | 1 | 1 | 1 | 1 | 1 |
| Cladocera | clad | 0 | 0 | 1 | 1 | 1 | 0 | 1 | clad | 1 | 1 | 1 | 0 | 1 | 1 | 1 | 1 | 1 |
| Coleoptera, Carabaidae | cara | 0 | 0 | 0 | 0 | 0 | 1 | 0 | cara | 1 | 0 | 0 | 0 | 1 | 0 | 0 | 0 | 0 |
| Coleoptera, Chrysomelidae | chry | 0 | 0 | 0 | 0 | 0 | 1 | 0 | NA |  |  |  |  |  |  |  |  |  |
| Coleoptera, Curculionidae | NA |  |  |  |  |  |  |  | curc | 0 | 0 | 0 | 0 | 0 | 1 | 1 | 1 | 1 |
| Coleoptera, Dryopidae | dryo | 0 | 1 | 0 | 0 | 0 | 0 | 1 | NA |  |  |  |  |  |  |  |  |  |
| Coleoptera, Dytiscidae | dytis | 1 | 1 | 1 | 1 | 1 | 1 | 1 | dytis | 1 | 1 | 1 | 1 | 1 | 1 | 1 | 1 | 1 |
| Coleoptera, Elmidae | NA |  |  |  |  |  |  |  | elmi | 0 | 0 | 0 | 0 | 0 | 0 | 0 | 1 | 0 |
| Coleoptera, Gyrinidae | gyri | 0 | 0 | 0 | 0 | 1 | 0 | 0 | gyri | 1 | 0 | 1 | 0 | 1 | 1 | 1 | 1 | 1 |
| Coleoptera, Haliplidae | hali | 0 | 0 | 0 | 1 | 1 | 1 | 1 | hali | 0 | 0 | 0 | 0 | 1 | 1 | 1 | 1 | 1 |
| Coleoptera, Hydraenidae | hydra | 0 | 0 | 0 | 0 | 0 | 0 | 1 | hydr | 0 | 0 | 0 | 0 | 0 | 1 | 0 | 0 | 0 |
| Coleoptera, Hydrophilidae | hych | 0 | 0 | 0 | 0 | 0 | 0 | 1 | hych | 1 | 1 | 0 | 1 | 1 | 1 | 1 | 1 | 1 |
| Coleoptera, Noteridae | NA |  |  |  |  |  |  |  | note | 0 | 0 | 0 | 0 | 0 | 0 | 0 | 1 | 0 |
| Coleoptera, Scirtidae | scir | 0 | 1 | 0 | 1 | 0 | 1 | 1 | NA |  |  |  |  |  |  |  |  |  |
| Collembola | NA |  |  |  |  |  |  |  | coll | 0 | 0 | 0 | 1 | 0 | 0 | 0 | 0 | 0 |
| Copepoda, copepods | cope | 1 | 1 | 1 | 1 | 1 | 1 | 1 | cope | 1 | 1 | 1 | 1 | 1 | 1 | 1 | 1 | 1 |
| Diptera, Ceratopogonidae | cera | 0 | 0 | 0 | 0 | 0 | 0 | 1 | cera | 0 | 0 | 0 | 0 | 1 | 0 | 0 | 0 | 0 |
| Diptera, Chaoboridae | chao | 0 | 0 | 0 | 0 | 1 | 0 | 0 | chao | 0 | 0 | 1 | 0 | 1 | 1 | 1 | 1 | 1 |
| Diptera, Chironomidae | chir | 1 | 1 | 1 | 1 | 1 | 1 | 1 | chir | 1 | 1 | 1 | 1 | 1 | 1 | 1 | 1 | 1 |
| Diptera, Culicidae | culi | 1 | 1 | 1 | 1 | 1 | 1 | 1 | culi | 1 | 1 | 1 | 1 | 1 | 1 | 1 | 1 | 1 |
| Diptera, Dixidae | NA |  |  |  |  |  |  |  | didix | 0 | 0 | 1 | 1 | 1 | 0 | 1 | 0 | 0 |
| Diptera, Stratiomyidae | stra | 0 | 0 | 0 | 0 | 1 | 1 | 1 | stra | 0 | 0 | 0 | 0 | 0 | 1 | 1 | 1 | 1 |
| Diptera, Tipulidae | NA |  |  |  |  |  |  |  | tipu | 0 | 0 | 0 | 0 | 0 | 0 | 0 | 1 | 1 |
| Ephemeroptera | NA |  |  |  |  |  |  |  | ephe | 0 | 0 | 0 | 0 | 1 | 1 | 1 | 1 | 1 |
| Ephemeroptera, Baetidae | baet | 1 | 0 | 0 | 1 | 1 | 0 | 0 | baet | 0 | 0 | 0 | 0 | 0 | 1 | 0 | 0 | 0 |
| Ephemeroptera, Caenidae | NA |  |  |  |  |  |  |  | caen | 0 | 0 | 0 | 0 | 0 | 1 | 0 | 0 | 0 |
| Ephemeroptera, Potamanthidae | NA |  |  |  |  |  |  |  | pota | 0 | 0 | 0 | 0 | 0 | 1 | 0 | 0 | 0 |
| Hemiptera, Belostomatidae | belo | 0 | 0 | 0 | 0 | 1 | 1 | 1 | belo | 0 | 0 | 0 | 0 | 1 | 1 | 1 | 1 | 1 |
| Hemiptera, Corixidae, Corixinae | cori | 0 | 0 | 1 | 1 | 1 | 1 | 1 | cori | 0 | 1 | 1 | 0 | 1 | 1 | 1 | 1 | 1 |
| Hemiptera, Gerridae | gerr | 1 | 0 | 0 | 1 | 1 | 0 | 0 | gerr | 1 | 0 | 1 | 0 | 1 | 1 | 1 | 1 | 1 |
| Hemiptera, Heteroptera, Reduviidae | NA |  |  |  |  |  |  |  | redu | 0 | 0 | 0 | 0 | 1 | 0 | 0 | 0 | 0 |
| Hemiptera, Hydrometridae | NA |  |  |  |  |  |  |  | hymt | 0 | 0 | 0 | 0 | 0 | 0 | 1 | 0 | 0 |
| Hemiptera, Mesoveliidae | meso | 0 | 0 | 0 | 0 | 0 | 1 | 1 | meso | 0 | 0 | 0 | 0 | 1 | 1 | 0 | 0 | 0 |
| Hemiptera, Nepidae | rafu | 0 | 0 | 0 | 0 | 0 | 0 | 1 | nepi | 0 | 0 | 0 | 0 | 1 | 1 | 1 | 1 | 1 |
| Hemiptera, Notonectidae | noto | 0 | 0 | 1 | 1 | 1 | 1 | 1 | noto | 0 | 1 | 1 | 1 | 1 | 1 | 1 | 1 | 1 |
| Hemiptera, Pleidae | NA |  |  |  |  |  |  |  | plei | 0 | 0 | 0 | 0 | 1 | 1 | 1 | 1 | 0 |
| Hemiptera, Saldidae | NA |  |  |  |  |  |  |  | sald | 0 | 0 | 0 | 0 | 0 | 1 | 0 | 0 | 0 |
| Hemiptera, Veliidae | micr | 1 | 0 | 0 | 1 | 1 | 1 | 1 | micr | 0 | 1 | 0 | 1 | 1 | 1 | 1 | 0 | 1 |
| Isopoda, Asellidae | asel | 0 | 0 | 1 | 1 | 0 | 0 | 0 | asel | 1 | 0 | 0 | 1 | 0 | 0 | 0 | 0 | 0 |
| Megaloptera, Corydalidae | mega | 0 | 0 | 1 | 1 | 1 | 1 | 1 | mega | 1 | 0 | 0 | 0 | 1 | 1 | 1 | 1 | 1 |
| Odonata (Anisoptera), Aeshnidae | aesh | 0 | 0 | 1 | 1 | 1 | 1 | 1 | aesh | 0 | 0 | 0 | 1 | 0 | 1 | 1 | 1 | 1 |
| Odonata (Anisoptera), Libullulidae | libu | 1 | 0 | 0 | 1 | 1 | 0 | 1 | libu | 1 | 1 | 1 | 0 | 1 | 1 | 1 | 1 | 1 |
| Odonata (Zygoptera), Coenagrionidae | coen | 0 | 0 | 0 | 1 | 1 | 1 | 1 | coen | 0 | 0 | 0 | 0 | 1 | 1 | 1 | 1 | 1 |
| Odonata (Zygoptera), Lestidae | lest | 0 | 0 | 0 | 1 | 1 | 1 | 1 | lest | 1 | 1 | 1 | 1 | 1 | 1 | 1 | 1 | 1 |
| Ostracoda | ostr | 1 | 1 | 0 | 1 | 1 | 1 | 1 | ostr | 1 | 0 | 0 | 1 | 0 | 0 | 0 | 1 | 0 |
| Trichoptera, Leptoceridae | NA |  |  |  |  |  |  |  | lept | 0 | 0 | 0 | 0 | 0 | 1 | 0 | 0 | 0 |
| Trichoptera, Limnephilidae | limn | 0 | 1 | 1 | 1 | 1 | 1 | 0 | limn | 1 | 1 | 1 | 1 | 1 | 1 | 1 | 1 | 0 |
| Trichoptera, Phryganeidae | NA |  |  |  |  |  |  |  | phry | 0 | 1 | 0 | 0 | 0 | 1 | 0 | 0 | 0 |
| Veneroida, Sphaeriidae | spha | 1 | 1 | 1 | 1 | 1 | 1 | 1 | spha | 1 | 1 | 0 | 1 | 1 | 1 | 1 | 1 | 1 |
