# Supplementary material for: Food Webs in Relation to Variation in the Environment and Species Assemblage: A Multivariate Approach
Source: PLoS One. 2015 Apr 16;10(4):e0122719. doi: 10.1371/journal.pone.0122719 (PMC4399920; doi:10.1371/journal.pone.0122719)
Supplement: S2 Table — (DOCX) [file pone.0122719.s006.docx]

S2 Table Supporting Information

S2 Table. Stable isotope baseline data used to calculate trophic position.

| Pond | Year | mean δ^15^N all basal sources | mean δ^13^C base1 = snails | mean δ^15^N base1 = snails | mean δ^13^C base2 = clams | mean δ^15^N base2 = clams | comments |
| --- | --- | --- | --- | --- | --- | --- | --- |
| Blue2 | 2008 | 1.97 | -34.02 | 3.15 | -30.25 | 3.52 |  |
| Indian | 2008 | -0.24 | -31.41 | 0.81 | -34.09 | 0.16 |  |
| P82 | 2008 | 0.75 | -34.77 | 1.30 | -32.31 | 2.18 |  |
| Qubs | 2008 | 0.19 | -30.36 | 0.52 | -29.57 | 0.90 |  |
| Link | 2008 | 0.91 | -30.93 | 2.45 | -31.99 | 3.89 |  |
| Winter1 | 2008 | -0.22 | -30.90 | 1.08 | -32.88 | 1.55 |  |
| Winter2 | 2008 | 0.58 | -32.07 | 1.55 | -27.87 | 1.72 | used pond means for base1 δ^13^C and δ^15^N |
| Snake | 2009 | -1.28 | -32.65 | 2.02 | -31.60 | 2.49 | used mean δ^13^C from all ponds for both snails and clams |
| Blue1 | 2009 | 2.04 | -35.19 | 2.82 | -31.60 | 2.49 | used mean from all ponds for clams |
| Indian | 2009 | 0.39 | -33.20 | 1.22 | -31.60 | 2.49 | used mean from all ponds for clams |
| Qubs | 2009 | 1.09 | -31.52 | 1.77 | -31.05 | 0.92 |  |
| P82 | 2009 | 1.05 | -34.25 | 0.80 | -33.11 | 2.46 |  |
| Link | 2009 | 1.45 | -32.91 | 2.82 | -31.89 | 2.98 |  |
| Winter1 | 2009 | 0.92 | -26.74 | 1.06 | -29.42 | 1.91 |  |
| Winter2 | 2009 | 1.44 | -32.65 | 2.02 | -30.26 | 2.20 | used snail δ^13^C mean from all ponds |
| Blue2 | 2009 | 2.70 | -34.98 | 3.67 | -34.10 | 4.45 |  |
